# Total Ossiculoplasty: Advantages of Two-Point Stabilization Technique

**DOI:** 10.1155/2012/346260

**Published:** 2012-08-16

**Authors:** Leonard Berenholz, John Burkey, William Lippy

**Affiliations:** The Lippy Group, 3893 E. Market Street, Warren, OH 44484, USA

## Abstract

*Objective*. Evaluate a porous polyethylene prosthesis with two-point stabilization in total ossiculoplasty. This approach utilizes a lateral as well as a medial graft to stabilize a total ossicular prosthesis (TOP). *Study Design*. Retrospective cohort review of total ossiculoplasty. *Methods*. All patients who underwent total ossiculoplasty during the years 2004–2007 were included in the study group. Only five patients (10%) had primary surgery whereas 45 (90%) underwent revision surgery. Cartilage grafts covering the prosthesis (Sheehy, Xomed) laterally were used in all patients with areolar tissue being used for medial stabilization at the stapes footplate. Follow-up examination and audiometrics were performed a mean of 8.1 months following surgery. *Results*. The percentage of patients closing their ABG to within 10 dB was 44% with 66% closing their ABG to within 20 dB. The mean four-frequency hearing gain was 15.7 dB. The mean postoperative ABG was 15.7 dB. *Conclusion*. Audiometric results following total ossiculoplasty surgery using two-point stabilization exceeded results from the otologic literature. Proper two-point fixation with areolar tissue and stabilization utilizing cartilage were the keys to achieving a relatively high percentage of success in chronic ear disease in this sample.

## 1. Introduction

Many developments in reconstruction of the ossicular chain have taken place over the last 50 years [1]. The biologic reconstruction efforts included both auto- and homografts. Autografts included bone chips from the mastoid cortex and ossicles usually consisting of a portion of the incus or malleus. Concern for reimplantation of cholesteatoma when using the incus eventually led to the decline in its popularity. In addition, the time involved in drilling the incus to modify the ossicle was seen as a disadvantage. Aside from the residual microscopic cholesteatoma disease, once modified, the malleus or incus might not be long enough, particularly in total ossiculoplasty [2]. Homografts were one of the first reconstructive options but later fell out of favor due to the increased resorption and possible infectious transmission [3]. Although these grafts could be treated with autoclaving or formaldehyde to eliminate the risk of cholesteatoma or infectious transmission, these processes created a greater burden in using homografts. Special storage requirements also increased the expense and led to decreased popularity. In a large review of homografts by Chiossone [4], the functional results were understood to be inferior to middle ear reconstruction undertaken with prostheses. In over 400 cases the incus was most commonly used with the malleus used nearly as often.

There has been a quest for the ideal middle ear implant with the understanding that the middle ear environment in chronic ear disease is probably the main factor in determining success [5, 6]. Over the last several decades there has been a shift from the dominant use of autografts [7, 8] to the use of prosthetics. Numerous technical advances have improved hearing results and long-term results. With the major innovation of utilizing cartilage as an interface between the prosthesis and the tympanic membrane, extrusions have been reduced. The alloplastic materials used have included Polycel, Plastipore, Bioglass, and Ceravital [9–14]. Other centers have favored hydroxyapatite [15–17] or titanium [18–22] as the complete implant. With numerous prostheses available, the otologist has a wide array from which to choose, but may find it difficult to know which middle ear implant works best. In the last 10 years, proponents of utilizing titanium as the ideal middle ear implant have reported their results in numerous reviews [18–22]. The advantages cited are numerous: lightweight, biocompatible, good sound transmission, MRI compatibility, and the visibility of the medial contact area of the prosthesis.

## 2. History

### 2.1. Autografts and Homografts

 Over 40 years ago, incus repositioning with homograft and autograft incus ossicles was first undertaken [7, 23, 24]. The early results were promising, with understanding that the grafts would become part of the host environment. Fusion of the bone graft to the malleus and stapes or footplate should achieve perpendicular action with good sound transmission [8]. However, it was later realized that these grafts could be of inadequate length and/or too wide for the narrow oval window niche. Displacement could occur at the footplate junction, or there could be bone-bone fusion laterally between the graft and the medial external auditory canal. Use of homografts requires special banks that might not be widely available.

### 2.2. Alloplastics: Proplast

The 1970s brought additional interest into trying to overcome the deficiencies of the autograft, homograft, and plastic implants of the 1950s and 1960s. Proplast, a combination of two polymers, had a number of advantages that could be utilized for middle ear reconstruction [9]. A high percentage of Proplast's volume is porous to allow for tissue integration and to prevent excessive host graft rejection. These pores in the Proplast material also allowed host fluids to infiltrate the prosthesis and facilitate the acceptance of the prosthesis. The unique problem with this prosthesis was its Teflon shaft, which was not amenable to contouring. A Teflon polymer, Proplast, had all the disadvantages of Teflon, particularly substantial reactivity in the middle ear [25]. 

### 2.3. Alloplastics: Plastipore

 A high-density polyethylene sponge, a machine-tooled form known as Plastipore, was then explored as an alternative and found to be a material that could be sculpted and shaped [13, 14, 26]. In addition, it had all of the advantages of Proplast. Plastipore and a thermal-fused form known as Polycel became the backbone for many prostheses. In spite of all the advantages of Plastipore, it was understood that the fibrous unions between the shaft and the footplate could also occur between the shaft and the promontory, Fallopian canal and scutum. This fixation to surrounding bone was a significant problem with homograft and allograft ossicles. Cartilage does not fix to surrounding bone and was considered as an option for columella replacement from tympanic membrane to footplate, but its lack of stiffness would be a significant disadvantage in physiologically transferring sound from the drum to the vestibule. Plastipore, being both rigid and easily sculpted, was understood to be an excellent alternative to autograft and homograft ossicles. The cartilage would interface between the tympanic membrane and the head of the prosthesis, both stabilizing it and reducing the risk of extrusion.

### 2.4. Polycel and Cartilage

 More than 20 years ago, Sheehy [13] further discussed the advantages of using cartilage under the tympanic membrane. Analyzing failures, he concluded that mucosal and tubal problems were responsible for the majority of extrusions. There was a higher incidence of extrusions in ears requiring a TOP, probably because of the more severe ear disease associated with a destroyed stapes superstructure. An additional modification to the TOP, adding Polycel, rendered the prosthesis even more tissue, tolerable while maintaining its elasticity and plasticity [11]. In significantly diseased ears, two stages appeared to increase overall success rate. Homograft ossicles were still used in 1987 due to their established history, but it was already clear that the functional hearing results were better with partial ossicular prostheses (POPs) and TOPs. Initial higher extrusion rates with the prosthetics significantly decreased with cartilage interface.

 Continued research in the late 1980s sought a more ideal material for the middle ear. Carbon matrix prostheses [27] were considered as ideal middle ear implants due to their relative nonreactivity and potential improved sound conductivity. Improved results with use of hydroxyapatite were reported by Black in 1990 [17]. No cartilage interface was used with the results that hearing in more diseased ears was significantly worse. In a review of cases that were performed over a 10-year period, Slater et al. [12] noted the advantages of porous polyethylene: ability to form a fibrous union, no need for the malleus, and decreased extrusion rate. The results of TOPs were not as good as POPs, theorized as being due to an unstable connection to the footplate. Dornhoffer, in 1998 [16], noted the ideal weight of a middle ear prosthesis and need to accommodate the malleus and minimize the angle formed with the tympanic membrane. The Dornhoffer prostheses are composed of hydroxyapatite and require preserving the tensor tendon.

### 2.5. Alloplastics: Hydroxyapatite

 Hydroxyapatite made its debut in the early 1980s; Grote, the first to use hydroxyapatite in the middle ear, found it compatible [28]. In a 2001 survey of otolaryngologists using middle ear prostheses, Goldenberg and Emmet [1] found that there was a significant increase in use of hydroxyapatite with high satisfaction. Hydroxyapatite is composed of calcium phosphate and is used in a dense or porous state. In a dense state it will bond to bone. It has been considered biocompatible in the ear, allowing it to be placed directly against the tympanic membrane. Because of its bone-like characteristics and affinity for bone, hydroxyapatite should not be placed close to the scutum. Following hydroxyapatite's introduction to middle ear surgery, there was a corresponding decrease in use of Plastipore and homograft bone. 

### 2.6. Titanium

 Titanium was introduced by a small number of US otolaryngologists in the late 1990s. In the mid-1990s, European otologists were the first to use titanium middle ear implants in significant numbers of patients [18]. Advantages cited were improved visibility via an open head, possible improved signal transfer at 2 kHz, improved handling to adjust to individual anatomy, and MRI compatibility [18, 19]. One noted difficulty with the total prosthesis was that it could not be naturally secured to the footplate, although the partial prosthesis could be coupled nicely to the stapes superstructure. A multicenter trial conducted by Krueger et al. in 2002 [19] evaluated both partial and total ossicular reconstructions with titanium. These patients all had well-ventilated middle ears and no history of mastoidectomy. The followup in this study was short (3 months) and with selection bias due to surgery performed only on healthy, well-ventilated middle ears. Gardner et al. [21] reviewed their initial results with titanium and felt that the results were significantly better than those obtained with hydroxyapatite. In particular, they cited improved visualization in the partial and total ossiculoplasties. In 2004, Martin and Harner [20] reviewed their experience with titanium and confirmed Dornhoffer's results with the Dornhoffer prostheses [16]. Specifically, hearing results were better in primary cases, partial ossiculoplasties, and intact canal wall versus canal wall down mastoidectomies.

### 2.7. Factors Affecting Success

 In 2001, Dornhoffer and Gardner [6] published an extensive review of chronic ear factors that might affect overall success postoperatively, specifically ossicular chain status, mucosal abnormalities, drainage, type of surgery, and revisions. The presence of the malleus was thought to be significant, although the difference was only 2.9 dB in total ossiculoplasties. Fibrotic mucosa generally predicted a worse overall result. Drainage was considered to be a negative factor as well as mastoidectomy, particularly canal wall down mastoidectomy. Revision surgery generated poorer hearing results as well.

In a review focusing on long-term results, Yung [5] noted continued decline in audiometric results 5 years postoperatively. This study included both partial and total ossiculoplasties with the majority being hydroxyapatite. He divided the late failures into disease-related and surgeon-related failures. The disease-related category included conditions due to fluid, fibrosis, and adhesion. Surgeon-related failures were attributed to difficulty anchoring the prostheses when the malleus and stapes superstructure were missing. In a recent review, Mishiro et al. observed deterioration of hearing improvement particularly in patients with cholesteatoma and/or atelectasis [29].

### 2.8. Two-Point Stabilization

Despite numerous papers suggesting titanium as the new best implant system, we elected to review our recent results with the porous polyethylene prosthesis (Plastipore) prior to changing our well-established technique that is based on the two-point fixation principle. In the total ossiculoplasty reconstruction, the lateral surface of the prosthesis is covered with native cartilage. This cartilage is placed just medial to the scutum in cases where the canal wall is intact and slightly lateral to the malleus if present. In canal wall down cases the cartilage is level with the facial canal superiorly and the remnant of the bony annulus posteriorly. The medial shaft of the prosthesis is centered over the footplate with a tissue graft interface. Our study focuses on surgical technique rather than on a discussion about which type of prosthesis is optimal. Emphasis on lateral coverage with cartilage is well documented in the literature as are numerous types of prostheses. What has received scant attention is what is happening at the medial side of the prosthesis and its stability at the footplate interface. Therefore, only one type of prosthesis was used in this study. Surgical success can then be associated more directly to the stabilization technique and not to choice of prosthesis type by limiting this as a confounding variable.

With the extensive history of ossiculoplasty over the last five decades in mind, the difficulties in more diseased ears and, in particular, in total ossiculoplasty, this study analyzes hearing results in a group of patients undergoing a method of total ossiculoplasty. The principle of stabilizing the total prosthesis with native tissue both at its medial and lateral end is the driving factor behind the two-point stabilization theory.

## 3. Materials and Methods

 There were 50 consecutive total ossiculoplasties performed over a two-and-a-half-year period (2004–2007). The age range was 5 to 79 years with a mean of 43.5 years (s.d. = 22.8 years). There were 22 males and 28 females. Primary surgery was performed in 5 (10%) and revision surgery in 45 (90%) patients. For 19 patients, it was the first revision; for 13 patients, the second revision; for 13 patients, the third or more revision. None of the revisions were second-stage ossiculoplasties. Indications for revision were infection, perforation, failed ossiculoplasty, and greater than 25 dB conductive hearing loss. The preoperative diagnosis was chronic otitis media in 18 (36%) patients. Conductive hearing loss was the preoperative diagnosis in 19 (38%) patients with cholesteatoma diagnosed in 12 (24%) patients and retraction in 1 (2%) patient. Fibrosis in the middle ear cleft was noted in seven patients (14%). Ventilation tube placement was required postoperatively in five patients (10%) with myringotomy performed in one patient. Indication for myringotomy and/or ventilation tube placement was development of serous otitis media postoperatively. In three patients, there was some degree of facial nerve prolapse and one with a very high jugular bulb, but this did not preclude reconstruction. One patient had cleft palate surgery in the past, and there was one patient with a congenital ear (incus and stapes missing). This patient had no history of chronic otitis media or cholesteatoma.

### 3.1. Audiometric Testing

 Subjects were tested pre- and postoperatively using standard audiometric procedures in double-walled sound rooms. Air conduction thresholds were measured at 250, 500, 1000, 2000, 3000, 4000, and 8000 Hz. Bone conduction testing was performed at 500, 1000, 2000, 3000, and 4000 Hz. Pure tone average (PTA) results were calculated using 500, 1000, 2000, and 3,000 Hz thresholds. Postoperative air-bone gap (ABG) was calculated by comparing the postoperative air conduction PTA to the postoperative bone conduction PTA. The speech reception threshold (SRT) was defined as the level (dB HL) at which the listener could identify spondee words 50% of the time. Speech discrimination was measured using taped W-22 25-word lists. Lists were generally presented at 30 to 40 dB SL. Masking was used in the nontest ear as needed. Postoperative testing was performed 2–23 months after surgery with a mean of 8.1 months.

### 3.2. Total Ossiculoplasty

The basic approach is the same in primary and revision surgery. Modification of the approach is used in canal wall down procedures. Almost all of our procedures are done under local anesthesia with intravenous sedation. Children under age 12 are operated under general anesthesia. If mucoid fluid is present, a ventilating tube may be placed at the end of the procedure. If the malleus is present, then the tensor tympani tendon is divided with a sharp instrument under direct vision or by palpation, allowing lateralization and enabling easier placement of the reconstruction prosthesis. From an incision above the auricle, loose areolar tissue is harvested. It is pressed in a fascia press and allowed to dry. Tragal cartilage is harvested if the surgery is transcanal. Alternatively, conchal cartilage is harvested if the ossicular reconstruction is being done during a mastoidectomy. The perichondrium is dissected off the cartilage and, if necessary, pressed and used to reinforce the tympanic membrane. The cartilage is sculpted in the shape of a dome to accommodate the tympanic membrane and the size of the posterior superior quadrant in the middle ear. The TOP is cut with a no. 15 blade, on a moistened tongue blade and, if the middle ear space is aerated, it is usually cut in half. In a canal wall down reconstruction or a shallow middle ear cleft, up to two-thirds of the length of the prosthesis will be removed. If there is a remnant of the stapes crura but not enough to support a POP, the crura may be lasered off with an argon laser in order to accommodate the TOP. This is done with 0.1-second exposure at a power of 1-2 watts. If there is an associated perforation, a temporalis fascia or scar graft is placed as an underlay initially. 

**Figure 1:**
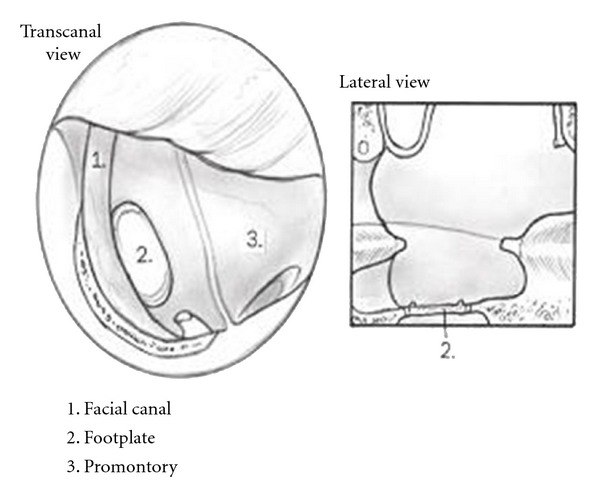
Surgical defect with mobile footplate requiring total ossiculoplasty.

**Figure 2:**
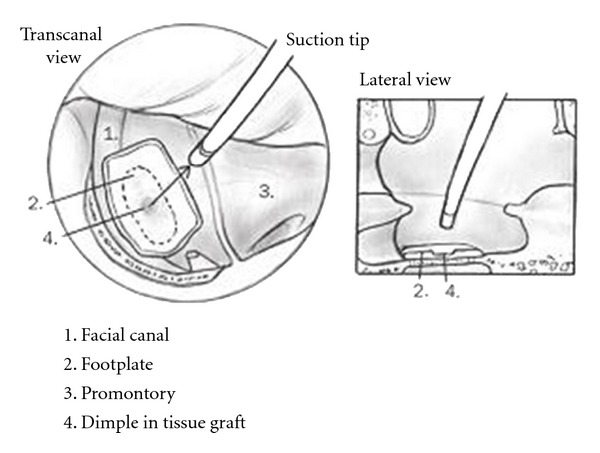
Areolar tissue graft placed over footplate and dimpled to receive TOP.

**Figure 3:**
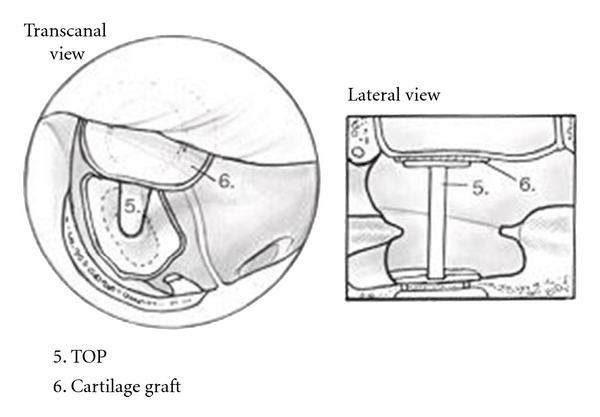
Lateral stabilization achieved with cartilage being placed lateral to TOP and medial to tympanic membrane.

Figures 1 through 3 demonstrate a step-by-step approach to achieve two-point stabilization utilizing areolar tissue and cartilage. First, the footplate is closely inspected to verify mobility (Figure 1). The mucoperiosteum around the footplate is abraded with a small hook to encourage adherence to the areolar tissue graft. The graft is trimmed to a diameter of approximately 3-4 mm so that it will cover the footplate and overlap slightly over the facial nerve and promontory. The graft is slightly rehydrated and placed using a cup forceps, then dimpled with a no. 24 suction in order to receive the TOP (Sheehy, Xomed) (Figure 2). Next, the TOP is placed over the areolar tissue and oriented in a perpendicular fashion. This is done using a no. 20 suction to stabilize the lateral disc part of the TOP and a footplate chisel to guide the medial shaft over the central portion of the areolar graft. Note that the areolar tissue helps self-center the TOP and prevents direct contact between the prostheses and the facial canal, footplate and promontory. The cartilage is placed lateral to the TOP and medial to the tympanic membrane (Figure 3). The lateral disc portion of the TOP rests under and medial to the central part of the sculpted cartilage. Gelfoam is not used in the middle ear unless an associated tympanoplasty is performed. The tympanomeatal flap is returned, and the cartilage elevated slightly to confirm prosthesis stability. In the reconstruction, the prosthesis is stabilized to avoid contact with the posterior bony canal wall. By sandwiching the TOP between the medial areolar graft and the lateral cartilage graft, stabilization of the TOP is enhanced and less likely to be displaced.

### 3.3. Intraoperative Audiometry

After the tympanomeatal flap has been returned and prior to placing gelfoam, intraoperative audiometry is performed. Those who did not receive intraoperative audiometry (26 patients) generally had a concomitant tympanoplasty or had a history of a canal wall down mastoidectomy.

 Intraoperative testing was performed using a Beltone 109 air-conduction audiometer with a TDH 39 headphone. The headphone was inserted into a Maico audiocup that fit around the ear and helped to attenuate the ambient noise that is common in a surgical suite. The sound pressure level measured with a Quest 155 precision sound level meter at the level of the ear was 54 dBA in our operating room with noise from background equipment and 30 dBA without equipment.

 A thickened tympanic membrane or perforation would contribute significantly to a conductive hearing loss; therefore no testing was done in these cases, nor was it done in seven children who were operated under general anesthesia. An orthopedic sleeve is used to keep the audiometer cable sterile. Preoperative air-conduction testing at 500 Hz is done in the operating room. This testing allows the surgeon to verify smaller improvements that routine tuning fork testing might not demonstrate. A positive Rinne will confirm closure of the ABG to within 25 dB, but intraoperative testing will verify more specific improvements. In seven cases, inadequate improvement in postoperative hearing prompted adjustment of the prosthesis with subsequent testing confirming improvement. Adjustments were made with a hook to better center the TOP over the central portion of the graft. Depending on the preoperative ABG, a moderate improvement (at least 10 dB) in pure tone thresholds at 500 Hz was expected.

The above testing is performed on patients sedated with the following protocol. In the holding area they are given Versed 2 mg. I.V. with Dramamine 50 mg orally. In the operating room they are given Fentanyl 100 mcg, Propofol 70–100 mg, and Zofran 4 mg all administered intravenously.

## 4. Results

The mean postoperative ABG was 15.7 dB (s.d. = 10.8 dB). The mean PTA hearing improvement was 15.7 dB (s.d. = 15.5 dB).  Table 1 shows the mean pre- and postoperative air and bone conductions as well as post-operative ABG.  Table 2 divides the patients into groups reflecting postoperative ABG. Closure to within 10 dB was achieved in 44% and to within 20 dB in 66% of patients. Sixty percent of patients benefited from greater than 11 dB improvement with 42% gaining more than 21 dB. Note that one patient developed a sensorineural drop 4 months after surgery, probably viral in origin. This patient had an MRI performed which was negative for a retrocochlear lesion. Microscopic examination revealed a moderate amount of adhesive otitis around the cartilage and TOP with no suggestion of an intrusion into the vestibule. There was no significant correlation between number of surgeries and postoperative ABG or hearing improvement (*r* = 0.19, *P* > 0.05).

Twelve patients in Group A (24%) had had a canal wall down mastoidectomy in the past with a postoperative ABG of 20.2 dB (s.d. = 10.4 dB) and mean hearing improvement of 11.8 dB (s.d. = 12.9 dB). Thirteen patients (26%) had an intact canal wall mastoidectomy in the past with a postoperative ABG of 14.8 dB (s.d. = 9.8 dB) and mean hearing improvement of 18.9 dB (s.d. = 14.9 dB). Neither the 7.1 dB difference in hearing improvement between these two groups (*t* = 1.05, *P* > 0.05) nor the 5.4 dB difference in ABG (*t* = 1.06, *P* > 0.05) was statistically significant. Twenty-five patients had total ossiculoplasty without history of mastoidectomy with postoperative ABG of 13.9 dB (s.d. = 11.3 dB) and a mean hearing improvement of 15.8 dB (s.d. = 17.1 dB). Comparing these 25 patients with those who had a mastoidectomy also failed to yield a significant difference between groups for hearing improvement (*t* = 0.07, *P* > 0.05) or ABG (*t* = 1.13, *P* > 0.05).

Intraoperative audiometry was performed in 24 of 50 of the total ossiculoplasties. Intraoperative testing was done only at one frequency, 500 Hz. The mean preoperative air conduction threshold at 500 Hz tested conventionally was 64.5 dB HL (s.d. = 23.2 dB) with intraoperative presurgery testing yielding 64.3 dB HL (s.d. = 18.4 dB). The mean conventional postoperative air-conduction threshold at 500 Hz. was 44.6 dB HL (s.d. = 22.8 dB) with postoperative intraoperative test yielding a mean of 40.1 dB HL (s.d. = 13.2 dB). There was only a mean 0.2 dB difference between preoperative intraoperative and conventional sound room audiometry. Postoperatively, the difference was 4.5 dB.  Table 3 compares 24 patients who had intraoperative audiometry performed with 26 patients who did not have intraoperative audiometry (IOA) done. Although there was no significant difference in ABG closure there was a significant difference (*P* = 0.016) in PTA improvement between those patients who had IOA and those who did not have IOA performed.

## 5. Discussion

 The challenge in ossicular reconstruction is well recognized. Certain variables such as middle ear fibrosis, adhesive otitis, and significant Eustachian tube dysfunction are not easily controlled by the otologic surgeon. However, two variables that can be controlled by the surgeon are the type of prosthesis used and the manner in which the prosthesis is used. The last several decades have seen a shift from autologous ossicle use to prosthetics [1]. Numerous implants have been developed during this period of time. Though not universally accepted, most otologists interface autologous cartilage between the prosthesis and the tympanic membrane [12]. Theoretically, interposition of tissue between the tympanic membrane and ossicular reconstruction might affect the hearing; this has been shown to be not significant [30]. Others have used hydroxyapatite directly under the tympanic membrane [31]. The last decade has brought titanium to the forefront. Numerous articles cite its advantages: tissue compatibility, durability, rigidness, lightweight features, and excellent acoustic transmission capability [18–22]. All titanium implants are MRI compatible.

 The focus of our review is to demonstrate the value of the two-point stabilization in total ossiculoplasty. As pointed out in the prior section on history, patients requiring a total ossiculoplasty generally have advanced disease. These patients typically arrive for reconstruction with a history of a number of procedures including intact canal wall and canal wall down mastoidectomy [32, 33]. All extrinsic and intrinsic factors must be controlled in order to optimize the chance for success in hearing restoration. Extrinsic factors such as associated nasal and sinus disorders should be treated prior to ossicular reconstruction. Intrinsic factors such as serous or mucoid effusion must be addressed intra- or postoperatively. Since the total ossiculoplasty is generally performed in a less than optimal physiological environment, the two-point stabilization concept is critical in maximizing the hearing result. Factors such as recurrent middle ear fluid, tympanic membrane retraction, fibrosis, and atelectasis of the tympanic membrane may displace the perfectly placed prosthesis over time. The shaft of the TOP needs be displaced only a fraction of a millimeter for it to adhere to the promontory and/or facial canal. The medial tissue interface of the areolar tissue graft may help to obviate this undesirable contact between the TOP and surrounding bony surface, hence avoiding prosthetic fixation.

The mean postoperative ABG of 15.7 dB and the finding that the ABG was closed to within 10 dB in 44% of patients are very favorable data for total ossiculoplasty.  Table 2 shows that 66% of patients closed their ABG to within 20 dB and 86% to within 30 dB. As shown in Table 4, no other review has demonstrated closure to within 10 dB as frequently as the current study, more impressive when one considers that almost 90% of the cases were revisions, almost half of which were second and third revisions. In addition, about one-quarter of the patients had a canal wall down mastoidectomy and another quarter had an intact canal wall mastoidectomy. In this group, 14% of patients had significant fibrosis, and an additional 10% required ventilation tube placement, both of which are factors that are recognized as having a potential adverse effect on the final hearing result. Several authors have noted the association between the above factors (revision, mastoidectomy, fibrosis, fluid) and a less successful result [6, 19]. In a multicenter study evaluating preliminary results with titanium, Krueger et al. [19] avoided prostheses in mastoid patients and chose well-aerated middle ears as implant candidates.

 Almost two-thirds (66%) of the patients in this study closed their ABG to within 20 dB (Table 2), and almost half the patients appreciated a 20 dB or greater PTA improvement in their hearing. There was no significant difference between primary and revision surgery outcomes. Noncanal wall down mastoidectomy (CWD) cases had ABG closure within 14.8 dB compared to 20 dB ABG closure for CWD cases. With slightly less hearing improvement and greater postoperative ABG, CWD cases did not do as well. There was one extrusion, this occurring in a case with subsequent adhesive otitis. A shorter prosthesis used initially might have prevented this.

 In addition, in a number of cases, intraoperative audiometry was very useful in verifying proper implant positioning. Intraoperative audiometry is a concept borrowed from otosclerosis surgery [34]. In this current paper, particular benefit was seen in cases in which we felt there should have been greater improvement during initial placement of the prosthesis. After readjustment of the prosthesis, repeat intraoperative testing confirmed better hearing in some cases.  Table 3 shows the significant difference in PTA improvement in the group that was tested intraoperatively. This group did have fewer tympanic membrane problems (thickening, adhesions, and perforations), and the PTA difference could possibly be due to this issue.

 Recent literature has confirmed that staging improves results particularly in more advanced chronic ears, especially those requiring total ossiculoplasty [35].

With different techniques and prostheses being used over the last 3 decades, it is difficult to compare studies. While recognizing that differences between studies in patient selection, technique and prosthesis type limit direct comparison of results, it is still interesting to contrast the audiometric results reported here to those of other studies. Table 4 compares the current study to five other studies of total ossiculoplasty. Comparison is made demonstrating audiometric outcomes including ABG closure and hearing improvement, duration followup, and numbers of patients. These results demonstrate the relatively high rate of ABG closure to within 10 dB in the study group. This table demonstrates the excellent results achieved with two-point stabilization. Table 4 compares the mean PTA ABG, mean PTA hearing improvement, and incidence of PTA ABG closure between studies. Regardless of whether we compare ABG, improved hearing, or incidence of ABG closure, the results for this study are very favorable. In addition, the variable pathology of chronic ear patients may not be comparable between studies unless strict criteria, such as those found in a middle ear risk index, are used [6]. In general, results are thought to be worse in mastoidectomy ears particularly canal wall down ears, and revision cases [6, 20]. These same authors confirmed poorer results with total ossiculoplasty due to the more severe underlying chronic ear disease.

The lateral stabilization over the prosthesis with cartilage has been well described [12]. What has not received attention is the medial stabilization of the shaft at the footplate area in total ossiculoplasty. The areolar tissue centers the shaft of the prosthesis and avoids a direct prosthesis-footplate bone contact. It decreases the risk of prosthesis-facial canal adhesion and prosthesis-promontory adhesion by interfacing soft tissue around it. Dimpling the center of the graft makes a total ossiculoplasty prostheses much easier to place and helps it to stabilize medially by self-centering. This concept is borrowed from the use of vein to cover the open vestibule once the footplate has been removed in stapedectomy [36]. The self-centering, native tissue interface between bone and prosthesis and medial shaft stabilization are the factors assisting in improved sound transmission. With 44% of patients closing their ABGs to within 10 dB and almost two-thirds to within 20 dB with medial stabilization, use of areolar tissue should be considered as part of the technical approach in total ossiculoplasty.

 The major weakness in this study is the retrospective nature, which is inherent in any review such as this. It would be of interest to compare various prostheses such as titanium, plastipore, and hydroxyapatite utilizing the two-point technique. Of course, a prospective matched cohort study with a larger sample size to increase the statistical power of the observations would lend more scientific support to this theory. Ideal comparisons in chronic ear studies with nonmastoid cases, primary and revision cases being compared only to each other, would further eliminate uncontrolled variables. Of interest would be comparisons of areolar tissue to vein, fascia, and perichondrium as the medial support. Areolar tissue was chosen because of its thinness as opposed to perichondrium and its easy availability.

 With the above in mind, further studies on large prospective groups with very well-controlled variables, using different prostheses with and without medial support as well as comparing various medial tissue grafts would be valuable. This study does not address long-term results in these difficult cases. Long-term review using this technique would perhaps show the value in two-point stabilization in obtaining more stable, prolonged reconstruction results.

In conclusion, we emphasize the two-point fixation principle in total ossiculoplasty reconstructions. Although this paper has focused on the porous polyethylene (Plastipore) prosthesis, two-point fixation may be achieved with all prostheses. The purpose of this study is not to propose one prosthesis over another. Rather, it is an attempt to overcome the difficult underlying conditions in total ossiculoplasty, particularly in mastoidectomy and revision ears. In total ossiculoplasty, areolar tissue over the footplate assists in the two-point stabilization. Following the above recommendations will maximize hearing improvement even in revision and difficult CWD reconstructions.

## Figures and Tables

**Table 1:** Mean pre- and postoperative air conduction (dBHL), bone conduction (dBHL), and word discrimination (%) results (Group A).

	250 Hz	500 Hz	1000 Hz	2000 Hz	3000 Hz	4000 Hz	8000 Hz	Discrim
Preoperative air	62.3	59.4	57.6	53.9	58.2	63.9	71.4	92.0
(S.D.)	(18.7)	(20.9)	(19.1)	(18.8)	(20.9)	(23.1)	(22.9)	(8.1)
Preoperative bone		23.9	22.1	30.2	30.4	31.6		
(S.D.)		(14.5)	(14.8)	(15.1)	(16.4)	(18.5)		
Postoperative air	45.6	43.2	39.2	36.6	47.5	55.4	66.4	91.7
(S.D.)	(22.4)	(23.3)	(22.4)	(21.0)	(23.1)	(25.0)	(25.9)	(16.3)
Postoperative bone		24.2	21.4	28.0	30.2	30.2		
(S.D.)		(16.3)	(17.4)	(19.0)	(19.6)	(21.2)		

**Table 2:** PTA air-bone gap (ABG) following surgery (Group A).

Postoperative ABG	No. of patients	% of patients
1–10 dB	22	44
11–20 dB	11	22
21–30 dB	10	20
31–40 dB	7	14

**Table 3:** Mean PTA air-bone gaps (ABG) and hearing improvement for patients who had and did not have intraoperative audiometry (IOA) during their surgical procedure (Group A).

	ABG	PTA improvement
24 patients with IOA	14.4 dB	21.1 dB
26 patients without IOA	16.9 dB	10.6 dB
Mean difference	2.5 dB	10.5 dB*

*This difference was significant (*P* = 0.016).

**Table 4:** Comparison of pure-tone average (PTA), air-bone gap (ABG), and hearing improvement results following total ossicular replacement.

	Current	Martin and Harner [20]	Gardner et al. [21]	Fisch et al. [22]	Krueger et al. [19]	Slater et al. [12]
Mean postoperaive ABG(dB)	15.7	25	24.6	21.2	15.8	NR
Mean PTA improvements(dB)	15.7	9	15.1	16.9	22.8	NR
% 0–10 dB ABG	44	3	7	13	26.7	38
% 0–20 dB ABG	66	40	44	57	66.7	67
PTA calculation	4 freq	4 freq	4 freq	4 freq	4 freq	3 freq
Mean followup	8.1 mo	3 mo–2.5 yr	1.5 yr	1 yr	3 mo	6 mo
*N*	50	30	27	46	15	133

NR: Not reported, mo: months, yr: years, *N*: number patients.
